# Elemental and Structural Characterization of Heterotopic Ossification during Achilles Tendon Healing Provides New Insights on the Formation Process

**DOI:** 10.1021/acsbiomaterials.4c00935

**Published:** 2024-07-23

**Authors:** Kunal Sharma, Isabella Silva Barreto, Hector Dejea, Malin Hammerman, Christian Appel, Kalotina Geraki, Pernilla Eliasson, Maria Pierantoni, Hanna Isaksson

**Affiliations:** † Department of Biomedical Engineering, Lund University, Box 118, 221 00 Lund, Sweden; ‡ MAX IV Laboratory, Lund University, 224 84 Lund, Sweden; § Department of Biomedical and Clinical Sciences, Linköping University, 581 83 Linköping, Sweden; ∥ Swiss Light Source, Paul Scherrer Institute, CH-5232 Villigen, Switzerland; ⊥ Diamond Light Source, Oxfordshire OX11 0DE, United Kingdom; # Department of Orthopaedics, Sahlgrenska University Hospital, 431 80 Mölndal, Sweden

**Keywords:** tissue characterization, X-ray fluorescence, small-angle X-ray scattering, wide-angle X-ray scattering, small animal model

## Abstract

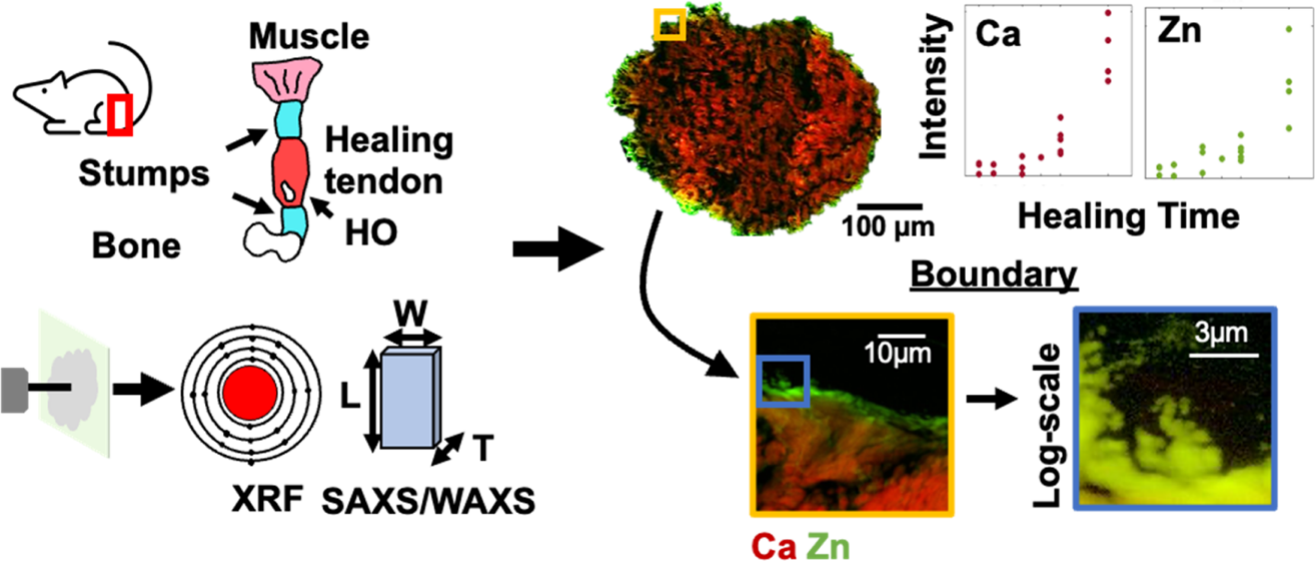

Heterotopic ossification (HO) in tendons can lead to increased pain and poor tendon function. Although it is believed to share some characteristics with bone, the structural and elemental compositions of HO deposits have not been fully elucidated. This study utilizes a multimodal and multiscale approach for structural and elemental characterization of HO deposits in healing rat Achilles tendons at 3, 6, 12, 16, and 20 weeks post transection. The microscale tomography and scanning electron microscopy results indicate increased mineral density and Ca/P ratio in the maturing HO deposits (12 and 20 weeks), when compared to the early time points (3 weeks). Visually, the mature HO deposits present microstructures similar to calcaneal bone. Through synchrotron-based X-ray scattering and fluorescence, the hydroxyapatite (HA) crystallites are shorter along the *c*-axis and become larger in the ab-plane with increasing healing time, while the HA crystal thickness remains within the reference values for bone. At the mineralization boundary, the overlap between high levels of calcium and prominent crystallite formation was outlined by the presence of zinc and iron. In the mature HO deposits, the calcium content was highest, and zinc was more present internally, which could be indicative of HO deposit remodeling. This study emphasizes the structural and elemental similarities between the calcaneal bone and HO deposits.

## 1 Introduction

Achilles tendon ruptures are common, and the healing process is complex, with no current consensus on optimal treatment after an acute injury.^1,2^ After rupture, tendons most often do not regain their original mechanical properties, resulting in reduced range of motion and load-bearing capability.^3^ Additionally, clinical studies reported radiographic “heterotopic ossification” (HO) in healing Achilles tendons.^4^ HO deposits are described as bone formation in soft tissue, where bone should normally not form;^5^ however, to our knowledge, it has not been directly compared at the nanostructural and elemental levels. The HO deposits may be associated with increased pain, impaired healing outcome, and increased re-rupture risk.^4,6,7^ One study reported 3 times higher incidence of HO in ruptured tendons compared to controls,^8^ and other studies have found that between 14–28% of patients developed HO after Achilles tendon repair.^6,9,10^ However, identifying the biological mechanisms is challenging in such a complex multiprocess environment. To provide a more controlled environment to study the process of HO after tendon injury, various animal models have been used.^11,12^ However, the mechanisms by which HO forms and progresses after tendon injury are still debated.

Our group recently showed that HO deposits also occur in intact rat Achilles tendons and that in transected tendons, they are present as early as after 1 week of healing.^13,14^ The volume of the HO deposits was found to substantially increase after 3 weeks of healing, reaching approximately 8% of the total tendon volume by 20 weeks of healing. The progression of HO in healing Achilles tendons started with deposits forming in the stumps, followed by ossification in the healing callus after 12 weeks of healing.^14^ Histological staining revealed regions of hypercellularity and high proteoglycan content potentially preceding ossification in areas where HO deposits were found at later time points.^14^ Similarly, another study observed abundant osteopontin by immunostaining in mice within the HO deposits,^15^ suggesting either bone-like structures or other forms of mineralized tissue.^16,17^ Finally, studies indicate that HO in tendons appear to occur through endochondral ossification,^5,18^ involving the initial deposition of a cartilaginous matrix that is later mineralized.^19^

From bone formation, it is known that the presence of proteins, such as matrix metalloproteinase (MMP) −9 and −13, play a pivotal role in remodeling the extracellular matrix with zinc (Zn) as a known cofactor.^20,21^ The presence of Zn at mineralization boundaries was shown in our previous studies using rodent models in long bone fracture healing^22^ and embryonic long bone development.^23^ The study on bone fracture healing also reported the presence of Iron (Fe) ahead of Zn at the mineralization front;^22^ however, the role that Fe plays at the mineralization front needs to be investigated further. From the literature, Fe can be linked to procollagen enzymes required for precursors in collagen.^24^ Furthermore, Fe was shown to accumulate at mineralization fronts,^22,24^ inside osteoblasts, and along edges of trabeculae structures.^25^ However, whether HO deposits in healing rat Achilles tendons share similar elemental characteristics has not been reported previously, to the best of our knowledge. Furthermore, the structural and elemental characteristics of HO in healing Achilles tendons have not yet been investigated.

A multimodal tissue characterization approach could yield insights into elemental and structural changes during deposition and progression of HO in healing Achilles tendons. In small animal models, microcomputed tomography (micro-CT) is the gold standard for visualizing and quantifying the internal structures and density of mineralized tissue. Additionally, scanning electron microscopy (SEM) allows one to investigate tissue micro- and submicron-structure changes and to correlate the structural features to elemental distribution by X-ray energy-dispersive spectroscopy (XEDS). Further hierarchical probing at the micrometer and nanometer scales can be achieved through synchrotron-based techniques.^26^ Small- and wide-angle X-ray scattering (SAXS/WAXS) techniques have been utilized extensively to investigate HA crystallite dimensions in bone.^22,23,27−32^ X-ray fluorescence (XRF) has been widely used to determine the elemental composition of mineralized tissues to elucidate mineralization pathways.^22,23,30,33,34^

This study utilizes a multimodal and multiscale approach for the same samples, with the aim to quantitatively characterize the formation of HO deposits in healing rat Achilles tendons and further elucidate the structural and elemental changes that occur during HO formation and maturation.

## 2 Methods

### 2.1 Animal Model

Animals used in this study were all part of a previous study where tendons from 29 rats undergoing healing were evaluated with high-resolution synchrotron-based phase contrast tomography.^14^ Representative samples were selected at time points where the percentual volume occupied by the HO deposits was steeply changing, from less than 1% (3 weeks) up to 8% (20 weeks) of the tendon volume.^14^ In this study, Achilles tendons from 14 female Sprague–Dawley rats (10–14 weeks old, weight 219 ± 21 g, Janvier, Le Genest-Saint-Isle, France) were surgically transected transversely and allowed to heal spontaneously.^35^ The rats were euthanized with carbon dioxide at 3, 6, 12, 16, and 20 weeks post injury, and their Achilles tendons, along with the calcaneal bone and muscle complex, were harvested. The rats were kept in pairs with a 12 h light/dark cycle at a temperature of 22 °C; food and water were provided ad libitum. The experiment adhered to institutional guidelines for the care and treatment of laboratory animals and was approved by the Regional Ethics Committee for animal experiments in Linköping, Sweden (Jordbruksverket, ID1424).

Before and after the phase contrast tomography,^14^ the samples were stored frozen until further processing. For the current study, the samples were fixed in formalin for 48 h, dehydrated, and further embedded in Poly/Bed 812 (DMP-30 Kit, Polysciences Europe GmbH). Embedded samples were imaged with micro-CT to localize the HO deposits. Subsequently, the embedded samples were sliced into 5 μm thin sections with a microtome (HM355S, Thermo Fisher Scientific, MA) and placed on Kapton tape (0.12 mm thickness, Micro to Nano, Netherlands). The number of animals per time point that were assessed with each technique are summarized in Table 1.

**Table 1:** Number of Animals Per Time Point Investigated with Each Experimental Setup^a^

	micro-CT	SEM	SAXS/XRF (10 × 20 μm^2^)	WAXS/XRF (2 × 2 μm^2^)	XRF (0.1 × 0.1 μm^2^)
3 weeks	*N* = 3	*N* = 1	*N* = 3	*N* = 3	*N* = 1
6 weeks	*N* = 2		*N* = 2		
12 weeks	*N* = 3	*N* = 1	*N* = 3	*N* = 2	*N* = 1
16 weeks	*N* = 1		*N* = 1		
20 weeks	*N* = 5	*N* = 1	*N* = 5		
bone	*N* = 4	*N* = 1	*N* = 2	*N* = 3	

a All samples were imaged with micro-CT to locate the HO in the embedded healing Achilles tendons. The same samples were used for the SAXS/XRF and WAXS/XRF experiments. Consecutive sections of one 3-week and one 12-week samples were used for nano-XRF. Beam spot sizes are listed for the synchrotron experiments. Samples from the calcaneal bone originated from the animals that were also included at other time points post-transection in this study.

### 2.2 Microcomputed Tomography (micro-CT)

All embedded samples were imaged with an EASYTOM150 (RX-Solutions, France) at 80 kV, 125 μA, with 1440 projections, and reconstructed to an isotropic voxel size of 19.1 μm to identify the presence and location of HO deposits. Phantoms with known hydroxyapatite (HA) densities (0.25 and 0.75 mg/mm^3^) were imaged to calibrate the grayscale values to mineral density (MD).

From the images, volumes of interest (VOI) were defined around the HO deposits to investigate the development of MD values across healing time points with custom MATLAB scripts. The threshold was set to 0.4 mg/mm^3^ to eliminate the embedding media. This value is below 0.42 mg/mm^3^, which is commonly used for low mineralizing tissue in bone research.^36^ The distribution of normalized MD was used to assess heterogeneity within each HO deposit and its progression across healing time points.

### 2.3 Scanning Electron Microscopy (SEM)

SEM was used to investigate the microstructure of HO deposits at different time points during the tendon healing process. HO deposits from 3, 12, and 20 weeks post tendon transection and a calcaneal bone comparison were used (Table 1). Block faces of samples were ground with SiC foil (Struers ApS, Sweden) with increasing grit size (800, 1000, 1200, 2000, and 4000) and were thereafter carbon coated (75 nm, BLAZERS CED030 carbon evaporator), mounted onto SEM stages with adhesive Carbon tape, and imaged with a JSM-6700F (accelerating voltage 20 kV, and 10 mA current). Images were acquired with backscattered electrons (BSE) at a working distance of 9.7 mm. Elemental analysis was carried out with X-ray energy-dispersive spectroscopy (XEDS) software AztecLive (Oxford Instruments) to calculate the Ca/P ratio. The regions containing HO deposits within each sample were exposed for 30 min to allow for a quantitative comparison.

### 2.4 Small-Angle X-ray Scattering and X-ray Fluorescence (SAXS and XRF)

To obtain the structural and elemental characteristics of a larger volume within the tendon, simultaneous SAXS and XRF measurements were performed at the cSAXS beamline (Swiss Light Source, Switzerland) with an X-ray energy of 12.4 keV, 10 × 20 μm^2^ spot and step size, and with a 300 ms exposure time. The SAXS detector (Pilatus 2M, Dectris, Switzerland) was placed at the end of a 7 m flight tube with a recorded sample–detector distance of 7.0982 m. The XRF detector (FalconX, XIA LLC) was placed ∼70 mm away at an angle of 50° with respect to the beam direction. Silver behenate powder standard was measured to calibrate the sample–detector distance and the beam center for SAXS analysis. Platinum (Pt) and Iron (Fe) standards were scanned for XRF calibration. Empty Kapton was scanned for background subtraction. All sample sections (*n* = 14) and calcaneal bone references (*n* = 4) were mounted on a custom-built aluminum plate, allowing for batch scanning with predefined regions. Regions containing HO deposits were identified using a calibrated optical microscope (IDS Imagining Development Systems GmbH, Germany, field of view of 1.8 mm).

The SAXS scattering patterns were analyzed after masking away the beam stop and hot/dead pixels and azimuthally integrated to generate *I*(*q*) curves for each data point. The HA platelet thickness (thickness, *T*) was calculated through iterative nonlinear least-squares curve fitting within the *q*-region (0.32–1.40 nm^–1^), under the assumption that the platelet shape has a finite thickness and two infinite dimensions, as previously described.^30^ Degree of orientation of HA crystals was determined through the ratio of anisotropic scattering and total scattering.^37^ The analysis was done with in-house MATLAB (R2020b, The MathWorks Inc.) scripts.^38^ For each pixel, XRF spectra were collected and fitted to quantify calcium (Ca) and Zinc (Zn). Structural and elemental distributions were averaged for each sample, normalized by sample area, and compiled to illustrate the structural and elemental characteristics of HO deposits at different healing time points.

### 2.5 Microprobe X-ray Fluorescence and Wide-Angle X-ray Scattering (Micro-XRF and WAXS)

To resolve the structural and elemental characteristics internally in individual HO deposits, micro-WAXS and XRF measurements were performed at the I18 beamline (Diamond Light Source, United Kingdom)^39^ with an X-ray energy of 12.0 keV, 2.5 × 2 μm^2^ spot size, 2 × 2 μm^2^ step size, and 300 ms exposure time. The WAXS detector (EXCALIBUR, 115 × 100 mm, 3-module)^40^ was placed ∼180 mm behind the sample stage, and the two 4-element XRF detectors (Vortex-ME4, Hitachi High-Tech Corp.) were placed ∼40 mm away at an angle of 20° with respect to the sample. Samples were mounted on custom holders and placed in the beam path at a 20 ^o^ offset angle. Background correction was performed based on measurements of Kapton alone within each sample. Samples from 3- (*n* = 3) and 12-week (*n* = 2) healing time points and calcaneal bone (*n* = 3) were selected (Table 1) from the same sections as the previous SAXS/XRF experiment, in order to represent the early and late time points of the study with enough repetitions within the time constraints of the allocated experimental time. From our previous study, it was observed that after 12 weeks, both the HO volume and density steeply increased.^14^ Therefore, the 12-week samples were selected to study the corresponding structural and elemental distributions in detail during a period of high HO deposit formation.

Analysis was carried out with custom beamline software DAWN.^41^ WAXS patterns were postprocessed by masking away the beam stop and azimuthally integrating the data to obtain 1D *I*(*q*) curves within the 1.5–3 nm^–1^*q*-range. The generated 1D *I*(*q*) curves were corrected for flux intensity variations with I0 measurements. The [002] and [310] reflections are related to the HA crystallite dimensions along the *c*-axis (length, *L*-parameter) and the ab-plane (width, *W*-parameter), respectively.^31,32,38^ The Scherrer equation was applied after 2 pixel × 2 pixel binning with in-house MATLAB scripts. XRF spectra were fitted, and elemental maps were generated using PyMCA.^42^

To analyze the distribution of trace elements and HA crystallite dimensions at the HO interface, distance maps were created by identifying the external boundary of the HO deposit and assigning each pixel a distance from the boundary. Resulting quantifications of Ca, Zn, Fe, and L- and W-parameters were averaged within samples and normalized by the maximum value across all samples. The combined distance, elemental, and structural maps were used to create line profiles across the mineralization boundaries. Analysis was performed with in-house MATLAB scripts.

### 2.6 Nanoprobe X-ray Fluorescence (Nano-XRF)

To obtain further in-depth information about the elemental distribution at the immediate mineralization front, nanoprobe XRF was utilized. Nano-XRF measurements were acquired at the NanoMAX beamline (MAX IV Laboratory, Sweden).^43^ Samples were scanned with an X-ray energy of 12 keV, 0.1 × 0.1 μm^2^ spot and step size and an exposure time of 50 ms. Measurements of AXO-C0 (Dresden GmbH) were acquired as calibration standards for XRF. Consecutive sections (5 μm) corresponding to previously imaged sections from two samples (3 and 12 weeks, *n* = 1 each) were mounted on silicon nitride (Si_3_N_4_) windows (1 μm thickness, 1 × 1 mm^2^ window, 4 × 4 mm^2^ frame, Silson Ltd., U.K.). These two samples were selected, as they appeared to be most active from the micro-XRF data of early and late time points.

Fitting and estimation of elemental concentrations was performed in PyMCA.^42^ Ca, Zn, and Fe concentration maps were analyzed at the HO boundary to further elucidate their role during active mineralization. All processing steps for micro- and nanoprobe results are available in Supporting Figure S1.

## 3 Results

### 3.1 Mineral Density and Microstructural Development of HO Deposits

**Figure 1:**
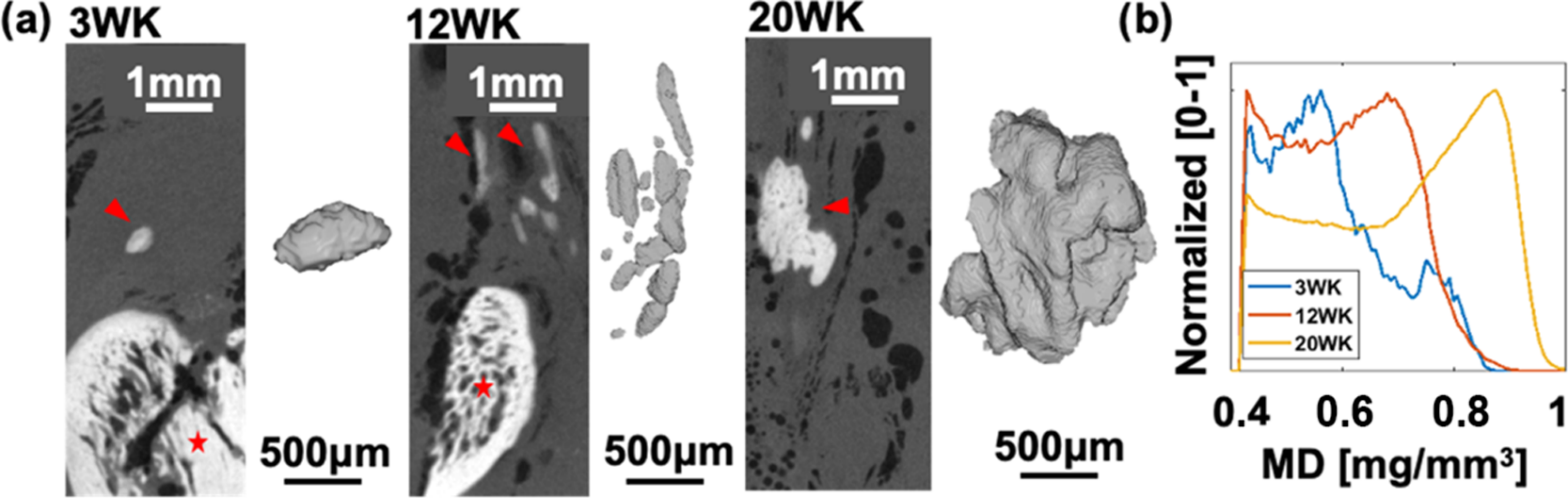
Visualization of HO deposits and mineralized density (MD) at different healing time points. (A) Micro-CT images with the HO deposit (arrowheads) and calcaneal bone (marked star), complemented with 3D volumes of the corresponding HO deposit. (B) Normalized distribution of mineral density averaged per time point (3, 12, and 20 weeks post injury).

The micro-CT images showed a clear increase in the volume of HO deposits at succeeding healing time points, thus indicating HO progression with time (Figure 1a), as we previously presented on a larger sample set.^14^ At 3 weeks post injury, the mineral density (MD) distribution presented two peaks, suggesting the presence of both low- and high-mineralized regions (Figure 1b, blue line). With time, the MD distribution progressed toward one peak in the high-mineralized region. The increased MD at 20 weeks post injury coincided with reduced heterogeneity (Figure 1b, yellow line), indicating increased maturation of the HO deposits.

The SEM images acquired showed different internal microstructures between the 3-, 12-, and 20-week samples (Supporting Figure S2). The XEDS measurements revealed that the average Ca/P ratios slightly increased at later healing time points (Ca/P ratios of 1.51, 1.69, and 1.79 for the samples after 3, 12, and 20 weeks post injury, respectively).

### 3.2 Structural and Elemental Characterization of HO Deposits

**Figure 2:**
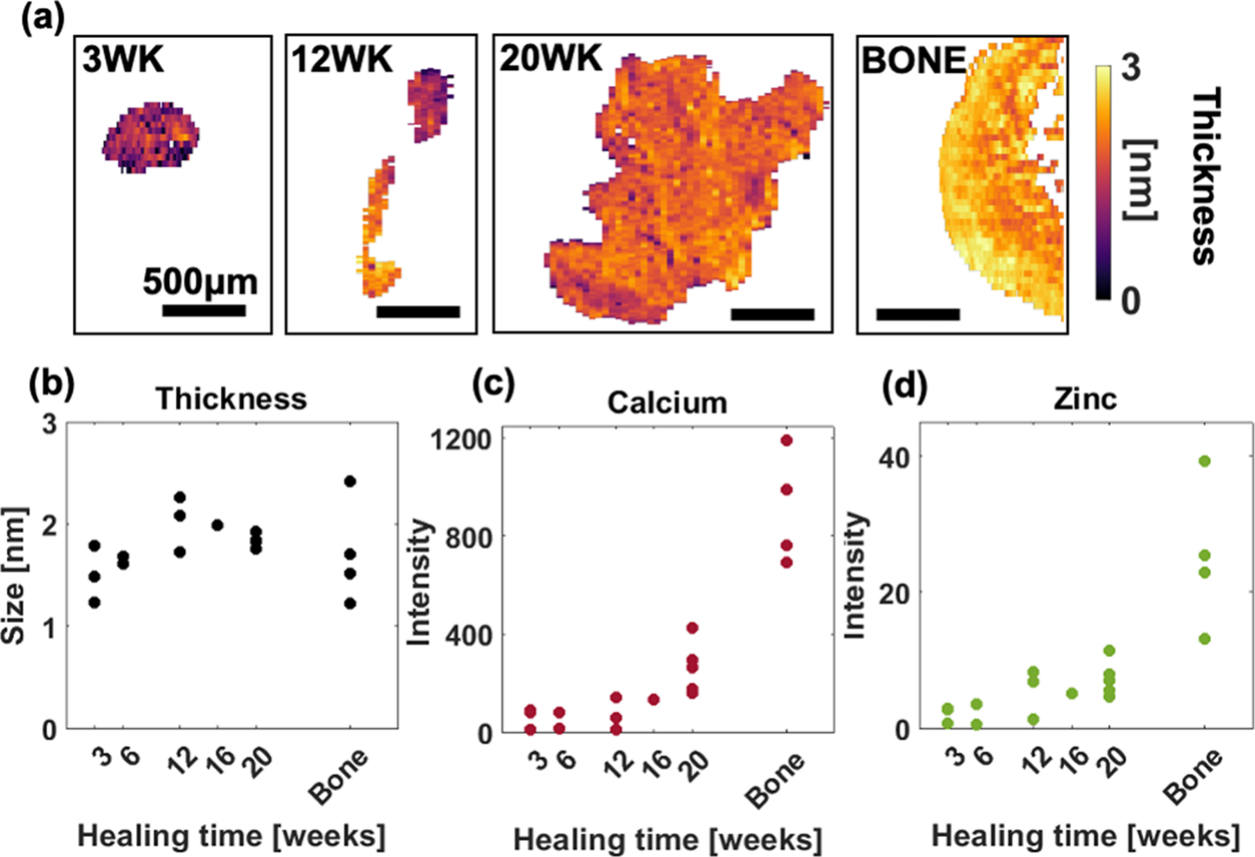
Global structural and elemental characterization of HO deposits at different healing time points. (a) HA platelet thickness visualized from SAXS data of HO deposits from 3, 12, and 20 weeks post transection and calcaneal bone (left to right). The average per sample across healing time points are presented for 3, 6, 12, 16, and 20 weeks post transection and bone reference for (b) thickness of HA, (c) calcium intensity, and (d) zinc intensity.

The average HA platelet thickness across the entire HO deposits increased slightly with healing time but generally stayed within 1.5–2.5 nm (Figure 2a,b). XRF measurements of entire HO deposits revealed that the concentrations of Ca and Zn were gradually increasing with healing time, without yet reaching the bone values at 20 weeks post transection (Figure 2c,d).

**Figure 3:**
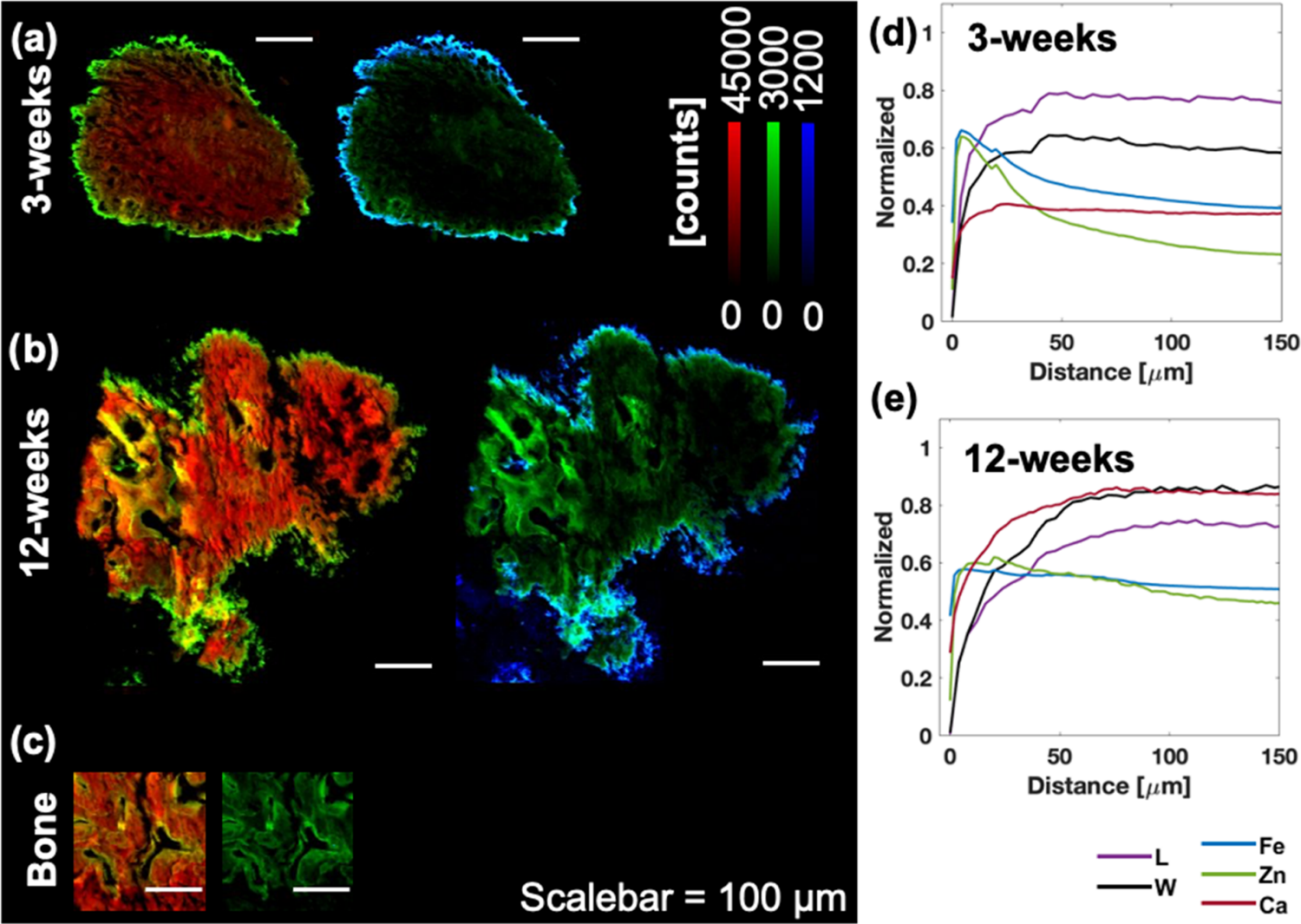
Simultaneous microprobe elemental and structural characterization of HO deposits. Combined XRF maps of Ca (red) and Zn (green), as well as Zn (green) and Fe (blue), highlight the elemental localization within the HO deposit for 3 weeks post injury (a), 12 weeks post injury, (b) and in bone (c). (d) and (e) illustrate the averaged and normalized presence of elemental (Ca, Zn, and Fe) and structural (*L*- and *W*-parameter) components at the HO boundary and with increasing distance (*x*-axis) toward the center of the HO deposit.

Simultaneous micro-XRF and WAXS measurements performed on 3 and 12 week samples allowed for a correlative analysis. Larger amounts of Zn were generally present at the boundaries of the HO deposits, although, at 12 weeks, high levels of Zn were also found internally along trabecular edges within the HO deposit (Figure 3a,b and Supporting Figure S3). A similar trend in which high Zn concentrations primarily surrounded internal microstructures was seen in the bone reference sample (Figure 3c). The intensity of Ca fluorescence was approximately 2-fold larger at 12 weeks compared to the deposits from 3 week samples (Figure 3a,b). The bone reference values reached approximately 2–3 times higher intensity for Ca fluorescence when compared to the HO deposits at 3 weeks (Supporting Figure S3).

To explore the elemental and structural changes at the mineralization front, line profiles were averaged and normalized for Ca, Zn, and Fe content, as well as for the structural *L*- and *W*-parameters. The resulting profiles at 3 weeks showed Zn and Fe intensity peaking prior to the plateau of Ca, *L*-, and *W*-parameters (Figure 3d). At 12 weeks, the profiles illustrated a more uniform distribution of Zn and Fe within the deposits (Figure 3e). Furthermore, the *L*-parameter was decreasing toward bone values, while the *W*-parameter appeared similar in the 3-week (80.9 Å) sample and bone reference (81.4 Å). However, after 12 weeks, the *W*-parameter was elevated, reaching 93.8 Å (Supporting Figure S4).

### 3.3 Nanoscale Elemental Characterization of HO Deposits

**Figure 4:**
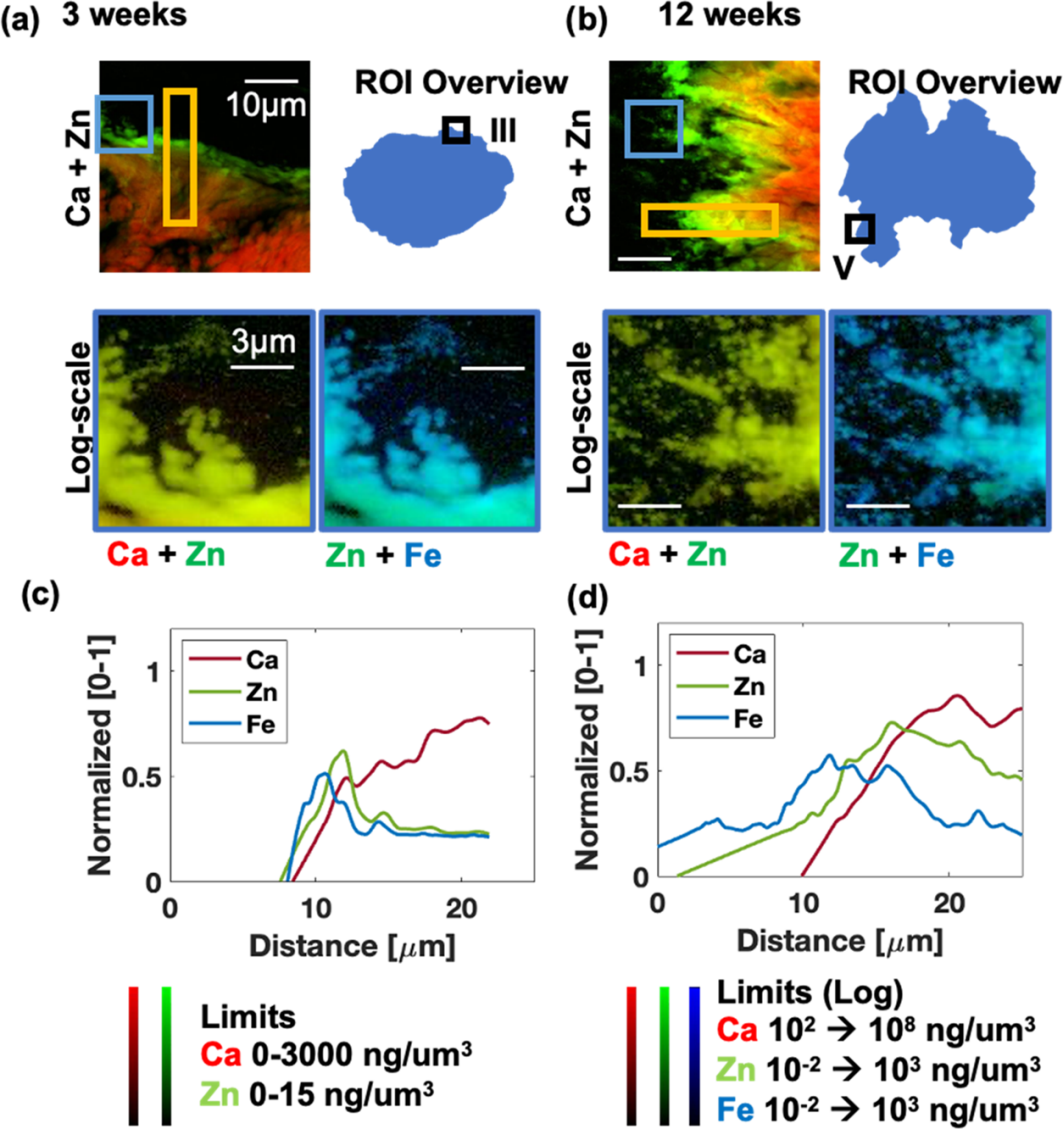
Nanoprobe XRF analysis of the HO boundary highlights the elements present in mineralization. Elemental maps of 40 × 40 μm^2^ of the HO boundary with Ca + Zn (top-left), ROI location (top-right), and log-scale (blue square indicates ROI, 10 × 10 μm^2^) combined Ca + Zn (bottom-left), Fe + Zn (bottom-right) for (a) 3 weeks, and (b) 12 weeks post injury. The ROIs for normalized and averaged line profile as determined through a 25 × 5 μm^2^ ROI (highlighted as yellow rectangles in Ca + Zn maps (a, b)) for 3 (c) and 12 weeks (d) post injury.

The nanoscale elemental results show large differences in internal structures at the boundaries of HO deposits when comparing between the HO deposits at 3 weeks and 12 weeks post injury. The boundary in the 3 week sample appeared well-defined, unlike in the 12 week sample (Figure 4a,b, top). From the log-scale visualization of the HO deposit in the 3 week sample, a few spherical particles (∼1–2 μm) were localized at the boundary (Figure 4a, blue box). Instead, the 12-week sample contained an extended mineralization front formed by elongated structures within which, in some cases, spherical particles were visible (Supporting Figures S5, and Figure 4b, blue box).

Zn and Fe distributions peaked at the boundary of the HO deposits in the 3 week sample, preceding the gradual increase of Ca further inside the HO deposit (Figure 4c). The HO deposit in the 12 week sample contained broader Zn and Fe peaks prior to the Ca increase (Figure 4d). Similar to the microscale results, higher total quantities (area under the curve) of all elements were observed in the HO deposit at 12 weeks post injury (Figure 4d).

## 4 Discussion

This study investigates the composition and structure at different length scales in the HO deposits that formed post injury in healing rat Achilles tendons at different healing time points through a multimodal tissue characterization approach. Mineral density continued to increase with increasing time after injury, suggesting HO maturation. This was also supported by SEM results, indicating higher Ca/P ratio values at later time points. The combined XRF and WAXS results correlate the presence of Ca within the HO deposits with trace elements such as Zn and Fe at the mineralization front, as well as their relation to crystallization seen through HA dimensions. This spatial correlation suggested that trace elements (Zn and Fe) play a part in the mineralization process preceding the mineral deposition and crystallization peaks (Figure 3d,e), and the nanoscale elemental results further indicated that Fe preceded Zn at the boundaries of HO deposits.

### 4.1 Differences in Internal Structures Suggest Two Different Types of HO Deposits

The mineral density of the HO deposits was on average higher at the later time point (20 weeks) when compared to the early healing time point (3 weeks). The increased mineral density, considered together with our previous findings, where the HO volume was seen to increase up to 8% of the tendon volume by 20 weeks post injury, indicates that deposits are both growing in size and also becoming more highly mineralized.^14^ Worth pointing out is that some HO deposits contained large internal variability in the mineral density. As an example, one 3 week sample contained two peaks in the normalized mineral density distribution, which, through micro-CT imaging, revealed two distinct internal structures. From the literature, it is known that one of the possible triggers for HO in tendons is inflammation resulting from injury.^44−47^ However, HO also occurs in intact rat tendons^13^ and physiologically in turkey^48^ and mouse^15^ tendons, suggesting that aside from injury and inflammation, there may be other factors at play. Furthermore, in our previous study, HO deposits with fiber-like internal structures were identified in intact rat Achilles tendons.^13^ A difference in the internal structure of the HO deposits was also seen in another study on healing rat Achilles tendons,^14^ suggesting the presence of areas of high MD within this 3 week HO deposit in the correspondence of the more fiber-like structure. Consequently, all of these observations together indicate that the two distinct peaks in MD could stem from an HO deposit present prior to injury (high MD), with surrounding regions of lower MD due to new mineralization occurring after injury.

### 4.2 Trace Elements at the HO Deposit Boundary

Through simultaneous micro-XRF and WAXS, the mineralization front of HO deposits was found to contain Zn and Fe, preceding high levels of Ca and crystallite parameters (*L*, along the *c*-axis; W, across the *ab*-plane). These results suggest that both Zn and Fe play roles in the HO formation process. Similar spatial distribution of Zn at the mineralization front has previously been described in mineralized tissues for rats and humans,^33,34,49,50^ and during embryonic development in mice.^23^ Zn is a known trace element with many functions, one of which is to stimulate osteoblasts while simultaneously inhibiting osteoclasts, thus allowing for bone growth.^51,52^ Higher quantities of Zn were observed in the 12 week samples, which coincided with a higher Ca/P ratio when compared to the early time point (3 weeks). From the literature, it is known that Zn is a cofactor that binds to alkaline phosphatase (ALP), which in turn creates a favorable environment for calcium phosphate (CaP) precipitation.^53^ Collagenase is also a Zn-dependent metalloenzyme used in the process of bone resorption and remodeling.^54,55^ In our study, Zn was found internally within the 12 week sample surrounding edges of trabecular structures, possibly suggesting the beginning of bone remodeling. Consequently, Zn could be a key player in multiple processes involved during HO, with this study providing a steppingstone for further elemental investigation of the formation pathways and remodeling. However, since Zn is a known trace element in both bone formation and prevention of bone resorption, further investigation is required to fully elucidate the role of Zn.

Furthermore, the crystallite dimensions in the 12-week sample obtained from micro-WAXS showed an increased *W*-parameter combined with a decreased *L*-parameter when compared to crystallite dimensions in the 3-week samples. Previous studies have found mineralization to occur along and across fibrils;^48^ this could explain the change in *L*- and *W*-parameters observed by micro-WAXS as the crystallite dimensions begin to expand in the *ab*-plane (*W*-parameter) while plateauing along the *c*-axis (*L*-parameter). This study observed larger dimensions (377–428 Å) along the crystallite *c*-axis, compared to the ab-plane (73–96 Å) appearing similar to a previously reported process where amorphous spheres progress toward a more elongated platelet.^56^ Overall, the simultaneous mapping of the elemental and structural properties of HO deposits allowed for a comprehensive view of the HO deposit characteristics and relationships among trace elements, mineral deposition, and crystallization.

From the nano-XRF data, we focused on the size and elemental composition of the particles that were present at the mineralization front. The nano-XRF results showed that Fe preceded Zn at all time points, suggesting that Fe also has an active role at the mineralization front of the HO deposits. The presence of Fe could be due to vascularization of the cartilaginous matrix^57,58^ or the result of cell death regulation by ferroptosis;^59^ however, this has, to our knowledge, not been previously reported for HO deposits. Further, our nano-XRF data showed Fe and Zn as spherical particles ahead of the Ca boundary, which are consistent in size (<300 nm in diameter) and shape with the reported^56^ spherulitic mineral foci found within fibrils and the surrounding extrafibrillar space in mouse bone.^60^ Spherulitic deposits of mineral (<500 nm) were also found between collagen fibrils at the mineralization front of HO in a murine Achilles tendon model.^15^ The nano-XRF results provided the possibility for further investigation by narrowing down the potential formation pathways where Fe is known to precede Zn during mineral deposition and crystallization.

### 4.3 Limitations

This study included samples from 14 animals that were selected from a previous study that quantified HO volume in 29 animals.^14^ Due to limited beamtime available at synchrotron facilities, unfortunately, a restricted number of samples could be studied with these high-resolution techniques. As the desired resolution directly affects the time required to scan a ROI, higher-resolution imaging was performed with a reduced number of samples compared to the lower-resolution investigations. The low sample numbers, particularly at higher resolution, therefore, did not allow for statistical analysis. However, the use of the same or consecutive sections for a multimodal and multiscale analysis allowed for a novel correlative investigation between multiple techniques, providing insights into the combined structural and elemental information about HO deposits. Unfortunately, as the tissue was processed with synchrotron-based techniques in mind, additional histological analysis of consecutive sections was not possible. However, we have previously presented histological data of HO deposits in healing rat Achilles tendons from the same animal model and at the same time points after injury.^14^

## 5 Conclusions

This study presented novel findings regarding the spatiotemporal structural and compositional characteristics of HO deposits in healing rat Achilles tendons. Maturation of the deposits was observed as increased mineral density, crystallite dimensions, and Ca content at late healing time points. For the first time, to our knowledge, the relative distributions of Ca, Zn, Fe, and HA crystallite dimensions were reported for HO deposits. The elemental analysis demonstrated elevated levels of Zn and Fe (with Fe preceding Zn) at the boundary of HO deposits, agreeing well with recent studies on ongoing mineralization in various bone models. Finally, accumulation of Zn was seen internally within the HO deposits at later time points, suggesting regions of higher activity of HA similar to known bone remodeling. These findings can provide further insight into the structural and elemental changes that occur during HO in tendons and serve as a stepping stone for further chemical evaluation of the role of trace elements such as Zn and Fe in the mineralization process.

## Supporting Information Available

Outlining the steps for processing SAXS, WAXS, and XRF data; microprobe XRF results for all 5 samples; microprobe WAXS results for all 5 samples; all nanoprobe XRF results with highlighted ROIs (PDF)
