# Supplementary material for: Elemental and Structural Characterization of Heterotopic Ossification during Achilles Tendon Healing Provides New Insights on the Formation Process
Source: ACS Biomater Sci Eng. 2024 Jul 23;10(8):4938–46. doi: 10.1021/acsbiomaterials.4c00935 (PMC11322912; doi:10.1021/acsbiomaterials.4c00935)
Supplement: Supplementary file 1 — ab4c00935_si_001.pdf [file ab4c00935_si_001.pdf]

Supplementary Material

Elemental and structural characterization of heterotopic ossification during Achilles tendon healing provides new insights on the formation process

Kunal Sharma[a], Isabella Silva Barreto[a], Hector Dejea[a,b], Malin Hammerman[a,c], Christian Appel[d], Kalotina Geraki[e], Pernilla Eliasson[c,f], Maria Pierantoni[a], Hanna Isaksson[a,*]

[a] Department of Biomedical Engineering, Lund University, Box 118, 221 00 Lund, Sweden

[b] MAX IV Laboratory, Lund University, 224 84 Lund, Sweden

[c] Department of Biomedical and Clinical Sciences, Linköping University, 581 83 Linköping Sweden

[d] Swiss Light Source, Paul Scherrer Institute, CH-5232 Villigen, Switzerland

[e] Diamond Light Source, Oxfordshire, OX11 0DE, United Kingdom

[f] Department of Orthopaedics, Sahlgrenska University Hospital, 431 80 Mölndal, Sweden

Pages: 5
Figures: 5

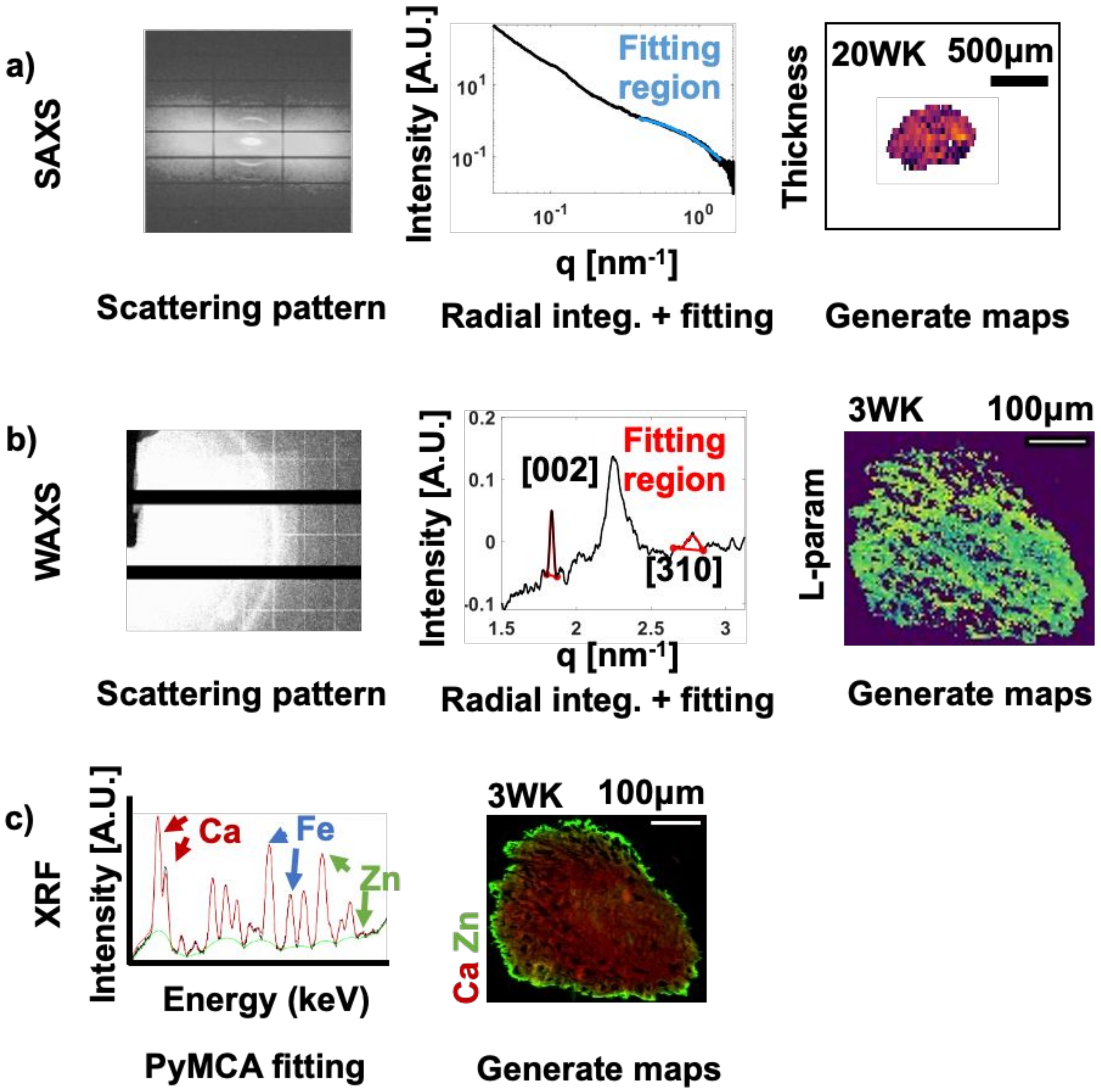

Figure S1: *Processing steps outlined for SAXS, WAXS, and XRF. SAXS (a) and WAXS (b) data analysis from scattering pattern, to I(q) curve fitting, and final resultant maps of quantified thickness (a), L- and W-parameters (b) of HA crystallites. XRF data analysis through PyMCA fitting and processing, to generated elemental maps (c)*

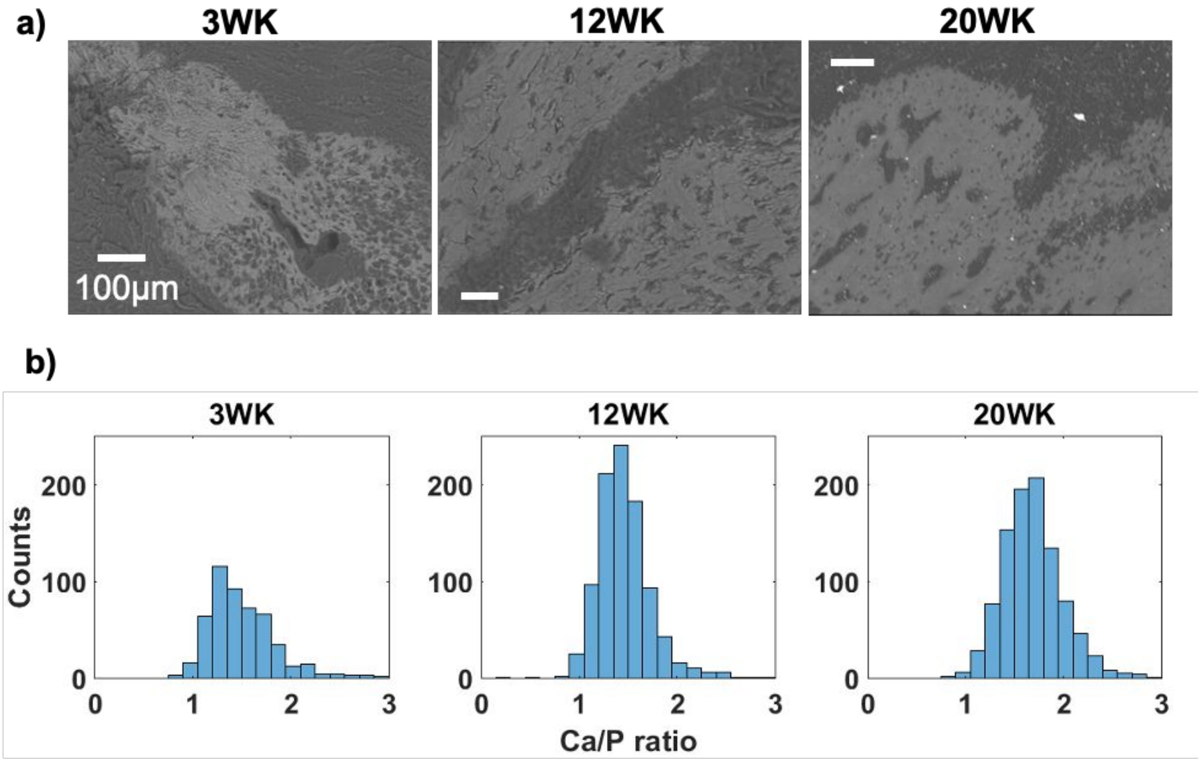

Figure S2*: SEM images of samples at 3-, 12-, and 20-weeks post injury showing a) internal structure differences at different healing time points, and the b) histograms for Ca/P ratios.*

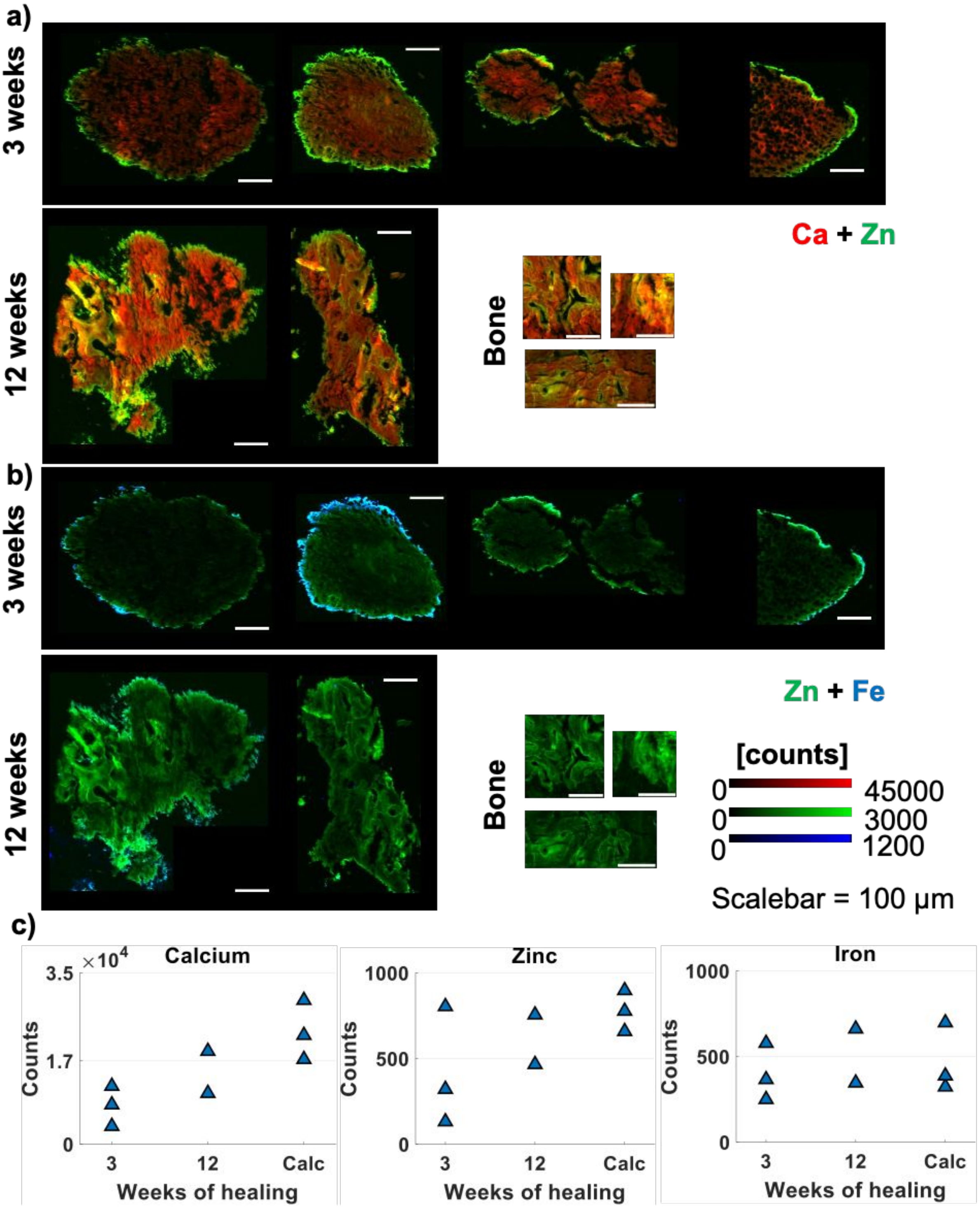

Figure S3. *XRF maps combining Ca+Zn and Zn+Fe localization in HO of healing Achilles tendons for 3- and 12-weeks post rupture.* (a) Ca+Zn maps for 3-weeks, 12-weeks post rupture, and for bone, (b) Zn+Fe maps for 3-weeks, 12-weeks post rupture, and for bone. (c) Averaged trends for HO per sample demonstrating the higher amount of Ca, and Zn at 12-weeks post rupture, while Fe levels remain constant.

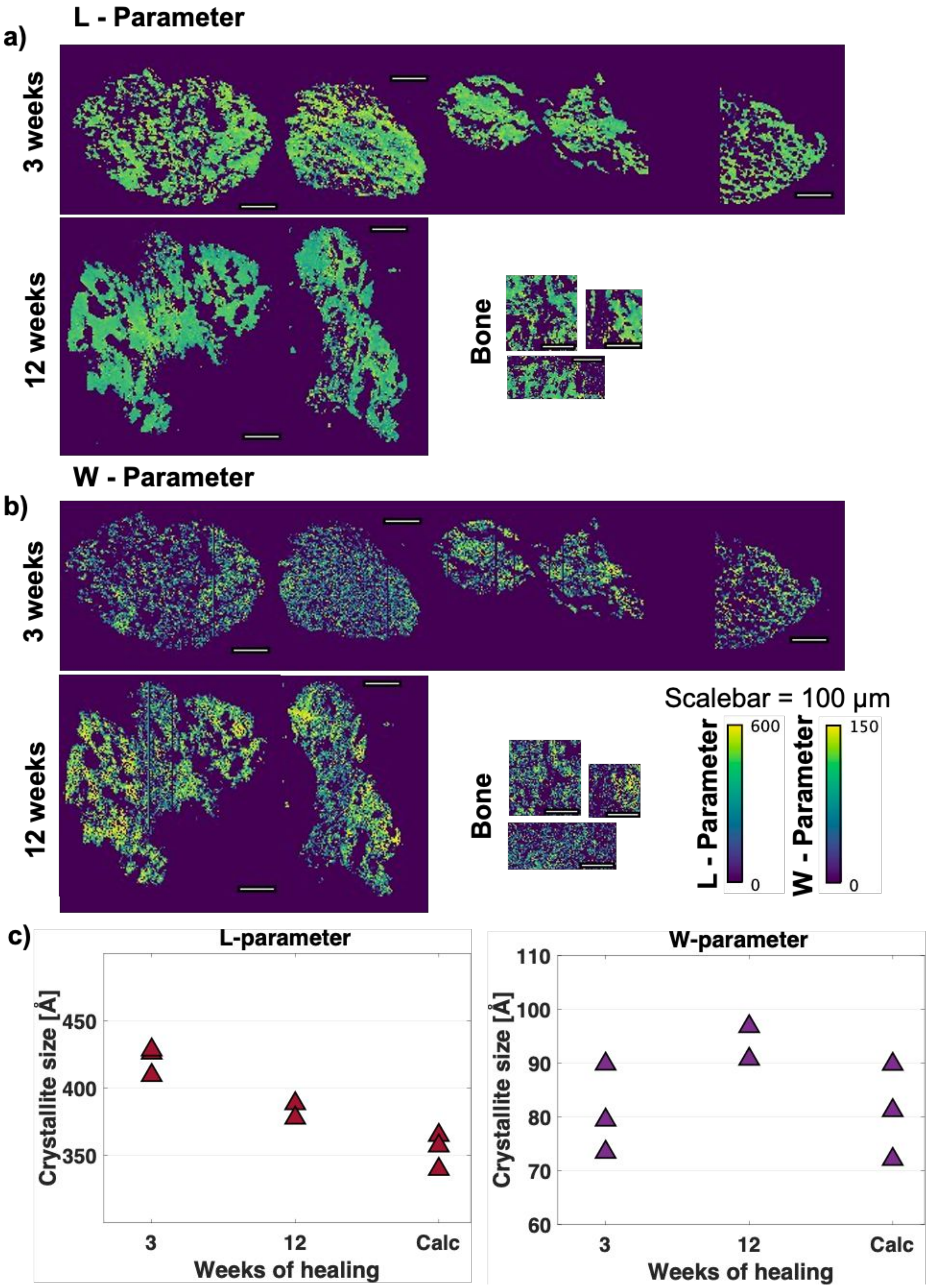

Figure S4. *XRD maps for L-parameter and W-parameter distribution in HO of healing Achilles tendons for 3- and 12-weeks post rupture.* (a) L-parameter maps for 3-weeks, 12-weeks post rupture, and for bone. (b) W-parameter maps for 3-weeks, 12-weeks post rupture, and for bone. (c) Averaged trends for HO per sample demonstrating the lower dimensions of the L-parameter across time points and bone, while at 12-weeks post rupture the W-parameter appears to be largest.

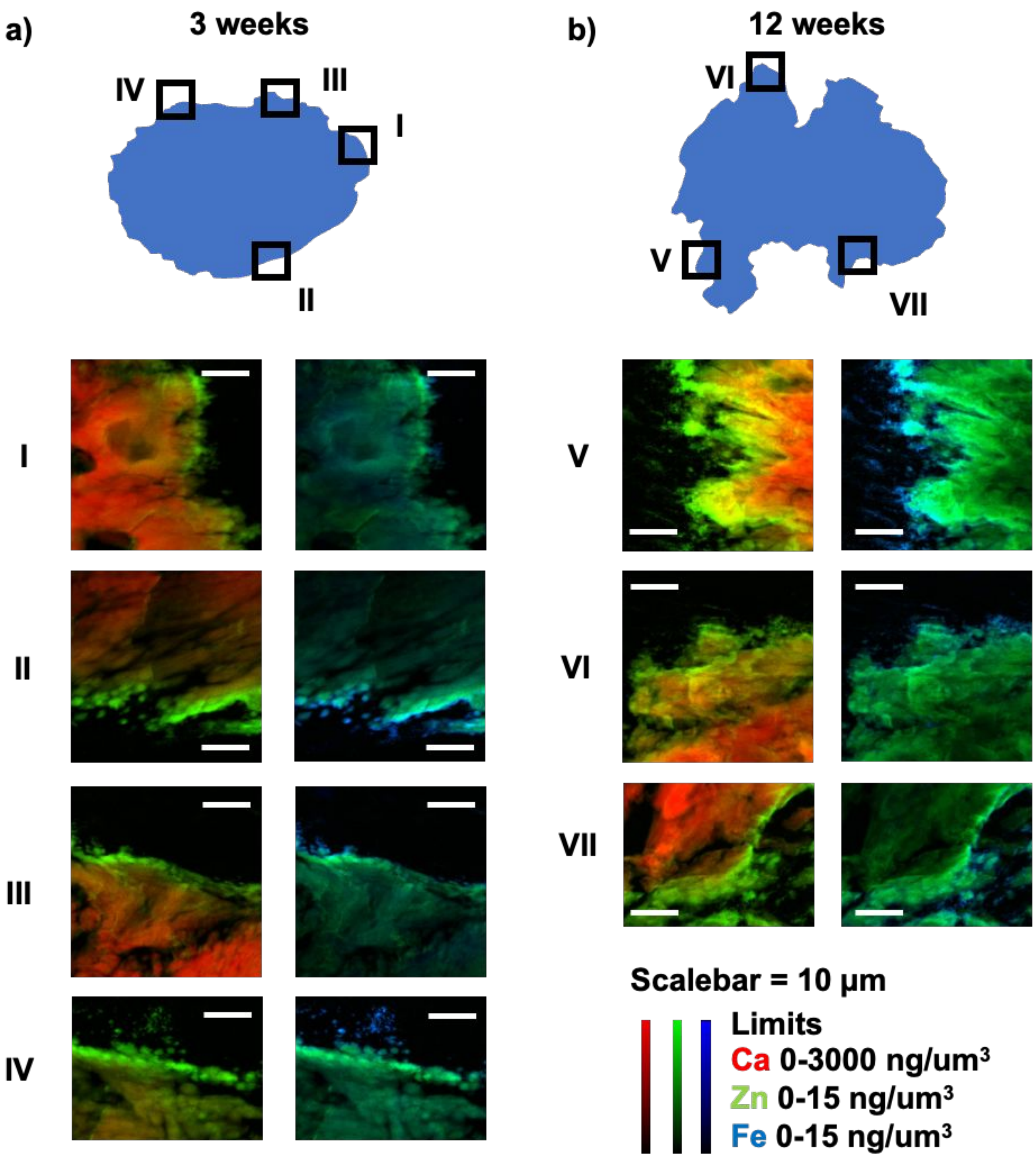

Figure S5. *Zoom in at the HO boundary, based on nanometer XRF. (a)* 3-week HO (HS218) with 4 different ROIs labelled on the overview schematic (top), the Ca+Zn maps (left) and the Zn+Fe maps (right) for each ROI (I-IV). (b) 12-week HO (HS71) with 3 different ROIs labelled on the overview schematic (top), the Ca+Zn maps (left) and the Zn+Fe maps (right) for each ROI (V-VII).
